# Lipocalin2 Promotes Invasion, Tumorigenicity and Gemcitabine Resistance in Pancreatic Ductal Adenocarcinoma

**DOI:** 10.1371/journal.pone.0046677

**Published:** 2012-10-04

**Authors:** Lisa Leung, Nikolina Radulovich, Chang-Qi Zhu, Shawna Organ, Bizhan Bandarchi, Melania Pintilie, Christine To, Devang Panchal, Ming Sound Tsao

**Affiliations:** 1 Ontario Cancer Institute/Princess Margaret Hospital, University Health Network, University of Toronto, Toronto, Ontario, Canada; 2 Department of Medical Biophysics, University of Toronto, Toronto, Ontario, Canada; 3 Department of Laboratory Medicine and Pathobiology, University of Toronto, Toronto, Ontario, Canada; Technische Universität München, Germany

## Abstract

Lipocalin 2 (LCN2) is a small secreted protein and its elevated expression has been observed in pancreatic as well as other cancer types. LCN2 has been reported to promote resistance to drug-induced apoptosis, enhance invasion through its physical association with matrix metalloproteinase-9, and promote *in vivo* tumor growth. LCN2 was found to be commonly expressed in patient PDAC samples and its pattern of immunohistochemical staining intensified with increasing severity in high-grade precursor lesions. Downregulation of LCN2 in two pancreatic ductal adenocarcinoma cell lines (BxPC3 and HPAF-II) with high LCN2 expression significantly reduced attachment, invasion, and tumour growth *in vivo*, but not proliferation or motility. Downregulation of LCN2 in two pancreatic ductal adenocarcinoma cell lines (BxPC3 and HPAF-II) with high expression significantly reduced attachment, invasion, and tumour growth *in vivo*. In contrast, LCN2 overexpression in PANC1, with low endogenous expression, significantly increased invasion, attachment, and enhanced tumor growth. Suppression of LCN2 in BxPC3 and HPAF-II cells increased their sensitivity to gemcitabine *in vitro*, and *in vivo* when BxPC3 was tested. Furthermore, LCN2 promotes expression of VEGF and HIF1A which contribute to enhanced vascularity. These overall results demonstrate that LCN2 plays an important role in the malignant progression of pancreatic ductal carcinoma and is a potential therapeutic target for this disease.

## Introduction

Pancreatic ductal adenocarcinoma (PDAC) is the fourth leading cause of cancer death in North America with an overall 5-year survival rate of <5% [1]. Previous PDAC microarray studies have revealed novel genes associated with disease progression. One of these was lipocalin-2 (LCN2), which was significantly overexpressed in PDAC cell lines and primary tumors compared to normal pancreas [2], [3]. LCN2 expression was also enhanced following KRAS oncogene expression in the normal human pancreatic duct epithelial cell line H6c7 [4].

LCN2 is also known as neutrophil gelatinase-associated lipocalin (NGAL) and belongs to a diverse family of lipocalins [5]. It binds covalently and non-covalently with a wide range of macromolecules including small hydrophobic ligands, soluble extracellular macromolecules, and iron [6]. Its expression is upregulated in epithelial cells under inflammatory conditions including appendicitis, organ damage, and pancreatitis [5], [7]. Overexpression of LCN2 has also been observed in a number of cancer types including breast, lung, ovary, thyroid, esophageal, and PDAC [8]–[12]. However, the precise role of LCN2 in cancer has not been completely defined. The covalent complex of LCN2 and MMP-9 has been associated with enhancing invasion and metastasis in breast cancer [12]–[14], poorer clinical outcome and improved migration in gastric cancer, [15], [16], and increased depth of tumour invasion in esophageal cancer [11]. In addition to its role in regulating MMP-9 activity, LCN2 has also been shown to promote cell survival in A549 and MCF-7 cells when treated with phosphoinositide-dependent kinase 1 (PDK1) inhibitors [17]. Its function in iron binding and transport has recently been shown to block the induction of the pro-apoptotic protein Bim and activation of caspase-9 which attenuates apoptosis [10]. The function of LCN2 in PDAC remains unclear. In this study, we examined the expression of LCN2 in precursor lesions of various grades and tumour tissue samples to correlate expression with the pathogenesis of PDAC. We also utilised tissue culture and mouse xenograft models to examine the function of LCN2 in PDAC. Here, we report that LCN2 contributes to the invasive, angiogenic, and drug resistant phenotypes in pancreatic cancer.

## Materials and Methods

### Cell Culture and in vitro Assays

Human PDAC cell lines, BxPC3, HPAF-II and PANC1 were obtained from the American Type Culture Collection (Manassas, VA). BxPC3 was cultured in RPMI media supplemented with 10% FBS. HPAF-II and PANC1 cells were cultured in DMEM media supplemented with 10% FBS. H6c7, H6c7 KRAS^G12V^, and H6c7KrT cell lines were generated as previously described [4]. Invasion assays were performed as previously described [18]. Adhesion assay: 100 000 (BxPC3, HPAF-II, and PANC1) or 250 000 cells (H6c7KrT) cells were seeded onto a 24-well dish coated with fibronectin and collagen (Sigma Aldrich, Mississauga, Canada) for 30 minutes (BxPC3, HPAF-II, and PANC1) or 45 minutes (H6c7KrT). The wells were stained with 0.2% crystal violet and lysed with 0.1% Triton X-100. The lysate was read at 590 nm on a Tecan XFlour4 plate reader (Mannedorf, Switzerland). Propidium iodide (Sigma Aldrich, St. Louis, MO) exclusion assay: 2.5×10^5^ cells were seeded on 6-well dishes treated with 10 µM gemcitabine or PBS for 72 hours. Live cells were resuspended in PBS supplemented with 0.5% BSA and 1 µg/ml PI, and analyzed by flow cytometry on a Becton Dickenson FACScan (Mississauga, Canada). To assess half maximal inhibitory concentrations (IC50), cells were grown in increasing concentrations of gemcitabine at 0, 0.001, 0.01, 0.1, 1, 5, 10, 20, 50, and 100 µM for 48 hours. Cell viability was assessed by MTS assay (Promega, Madison, WI). Wound healing assay: 2.5×10^5^ cells were seeded on 6-well dishes once cells and were serum starved upon confluence. The confluent cell layer was wounded and images were taken at 0, 24, and 48 hours to assess motility.

**Figure 1:**
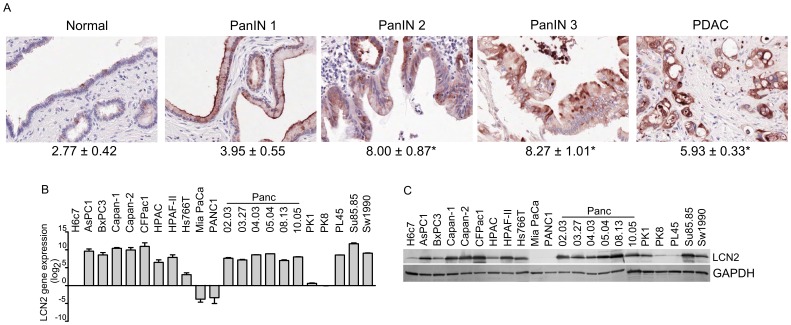
LCN2 expression in pancreatic neoplastic lesions and PDAC cell lines. (A) The LCN2 immunostaining pattern for normal (n = 31), PanIN1 (n = 22), PanIN-2 (n = 13), PanIN -3 and PDAC (n = 82). Mean scores and the SEM for LCN2 immunostaining are noted below the sections. (B) LCN2 gene expression was examined in 21 different PDAC cell lines. Relative expression was normalized using loading controls and then normalized to the H6c7 ratio. (C) Representative immunoblots of LCN2 and GAPDH protein expression in PDAC cell lines.

## Quantitative PCR

Total RNA isolation and PCR was performed as described before [18]. Primer sequences are provided in Table S4.

**Figure 2:**
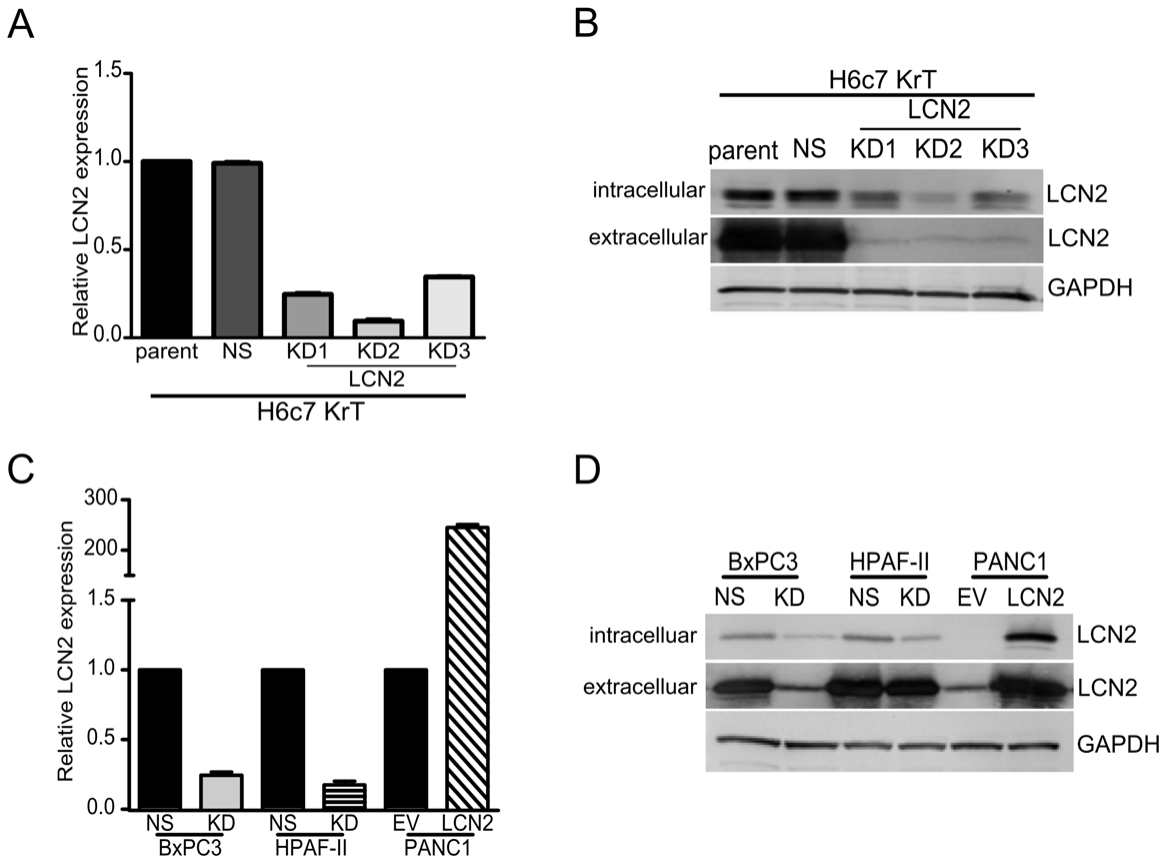
The knockdown and overexpression of LCN2 expression in PDAC cell lines. (A) LCN2 mRNA expression was suppressed using three different retroviral shRNA constructs (KD) in H6c7KrT cells. (B) Representative immunoblots of LCN2 protein expression in H6c7KrT cells, where GAPDH was used as a loading control. (C) LCN2 expression was downregulated in BxPC3 and HPAF-II cells. In PANC1 cells LCN2 was overexpressed by a lentiviral expression construct. (D) Representative immunoblots of LCN2 protein expression in BxPC3, HPAF-II, and PANC1 cells.

### LCN2 mRNA Silencing and Overexpression

LCN2 expression was stably downregulated by shRNA retroviral transduction method. The shRNA sequences were ligated into the pSUPER GFP retrovirus vector after linearization with Bgl*II* and Hind*III* (New England Biolabs, Pickering, Canada). The shRNA oligonucleotides used were: LCN2KD1: CGCATGCTATGGTGTTCTTCAA, LCN2KD2: AACCTCCGTCCTGTTTAGGAAA, LCN2KD3: GAGTTCACGCTGGGCAACATTA, and non-silencing control siRNA sequence: TTCTCCGAACGTGTCACGT (Qiagen, Dusseldorf, Germany). The shRNA retroviral expression vectors were generated using Phoenix-amphotropic packaging cell line (ATCC). The LCN2 expression construct was generated using our modified Gateway recombination cloning system [19]. LCN2 cDNA was reversed transcribed from the BxPC3 mRNA and amplified using the Platinum Pfx DNA polymerase (Invitrogen, Burlington, Canada) and gene specific primers (F: 5′CTGCCGCACCAGCCCGAAAGGCGCGCCT3′; R: 3′GACGGCGTGGTCGGGCTTTCCGCGCGGA5′). The amplicon was subcloned into an entry vector pENTR-CMVON plasmid after linearization with Asc*I* and Swa*I* restriction enzymes (New England Biolabs), which then underwent recombination with a lentiviral plko.YFP destination vector using LR Clonase II (Invitrogen). The resulting plko.YFP-LCN2 vector was stably transduced by lentiviruses.

**Figure 3:**
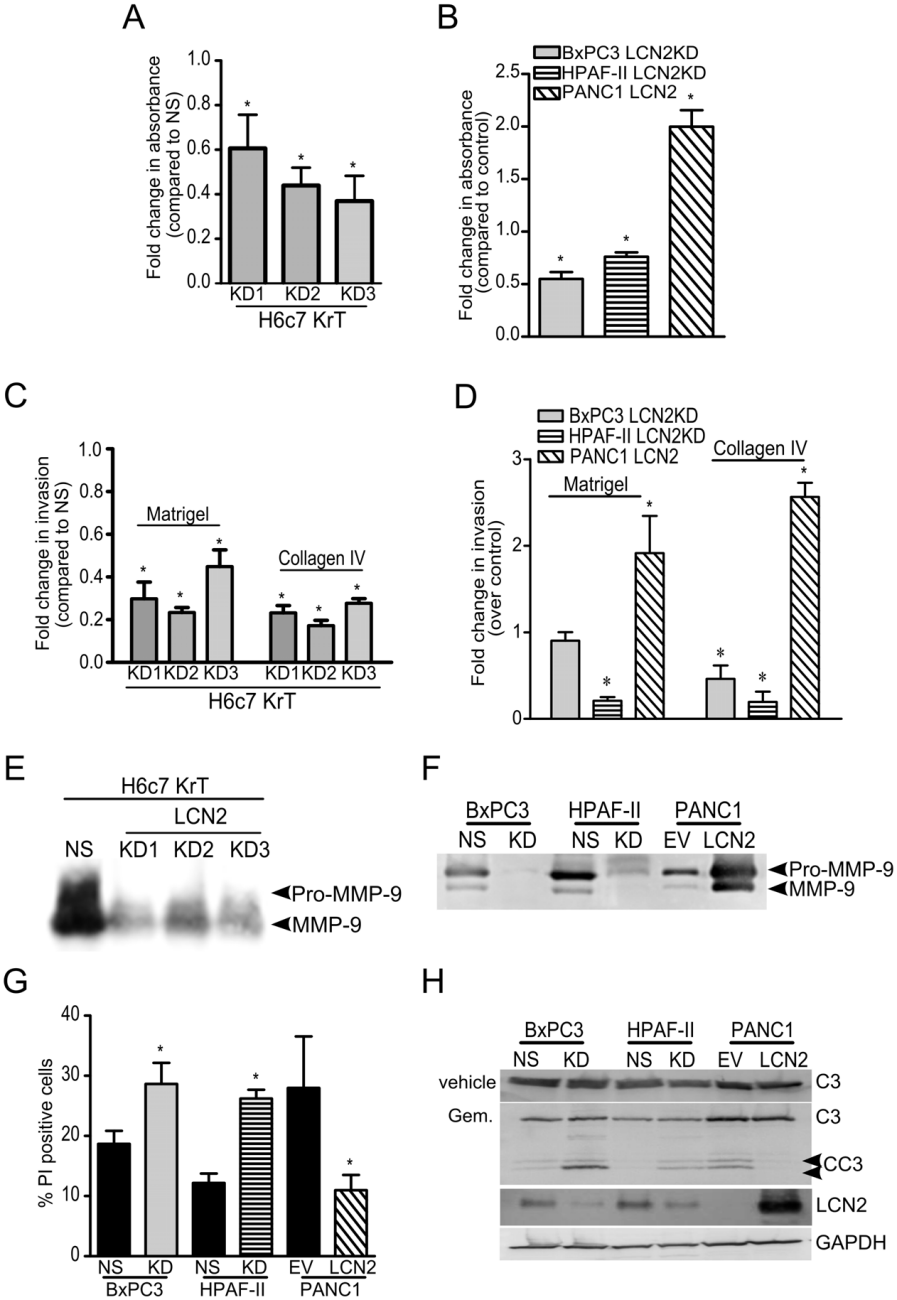
LCN2 promotes adhesion, invasion, and gemcitabine resistance in PDAC cells. Adhesion assays on the (A) H6c7 KrT, (B) BxPC3, HPAF-II, and PANC1 cell lines. Fold changes were calculated by comparing the KD to NS or LCN2 to EV (n = 3). The fold changes in invasive ability were calculated by comparing the effects of the shRNA constructs against the NS control, or LCN2 overexpression compared to the EV control. Invasive ability was assessed in (C) H6c7KrT cells (n = 3), (D) BxPC3, HPAF-II, and PANC1 cells (n = 6) were seeded onto Matrigel or collagen IV coated membranes. To assess MMP-9 activity gelatin zymography was performed on the conditioned media from (E) H6c7 KrT cells, (F) BxPC3, HPAF-II, and PANC1 cell lines (n = 3). (G) PI exclusion assays for cell death (n = 6) and (H) immunoblot analysis after 72 hours treatment by gemcitabine on the BxPC3, HPAF-II, and PANC1 cell lines (n = 3). (Gem., gemcitabine; C3, caspase 3; CC3, cleaved caspase 3; * denotes significant differences between the test and control samples student t-tests or one-way ANOVA and Bonferroni’s post hoc tests where appropriate.).

### Immunoblot and Gelatin Zymography

Immunoblotting was performed as described previously [18]. The primary antibodies used included total and cleaved caspase-3 (Asp175) and GAPDH (Cell Signaling, Danvers, MA); and LCN2 (catalogue no. AF1757, R&D Systems Minneapolis, MN). Visualization was accomplished by HRP-linked anti-rabbit, anti-mouse (Cell Signaling), and anti-goat secondary antibodies (Santa Cruz Biotechnology Inc, Santa Cruz, CA). Visualization was achieved by ECL-Plus detection kit (GE Healthcare, Baie d’Urfe, Canada) on a Typhoon phospho- and fluorescent imaging system 9400 (GE Healthcare). Gelatin zymography was performed on conditioned media and protein extracts from xenografts as previously described [20]. Resulting blots and gels were analyzed with ImageJ software (NIH, Bethesda, MA).

**Figure 4:**
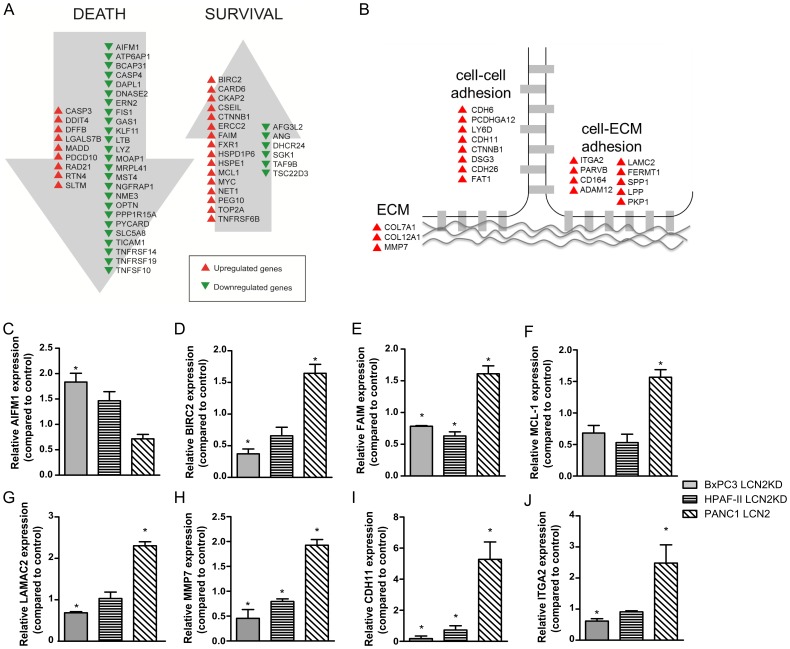
LCN2 promotes survival and adhesion. (A) LCN2 enhances the expression of anti-apoptotic genes and downregulated the pro-apoptotic genes. (B) LCN2 enhances adhesion and ECM. Target genes whose expression was up/downregulated by at least 1.5-fold in the control cell line and xenograft samples compared to the LCN2 downregulated cell line and xenograft samples. Red triangles denote increased expression and green triangles denote decreased expression. The mRNA expression of (C) AIFM, (D) BIRC2, (E) FAIM, (F) MCL-1, (G) LAMAC2, (H) MMP7, (I) CDH11, and (J) ITGA2 were assessed in BxPC3, HPAF-II, and PANC1 cell lines. (* denotes significant differences between the test and control samples (p<0.05, student t-tests, n = 3).

### Animal Work

All studies were conducted using protocols approved by the Ontario Cancer Institute Animal Care Committee (animal use protocol 745.09). Tumor growth and implantation was assessed as described before [18]. To assess gemcitabine sensitivity, each cell line was implanted subcutaneously into 20 mice. Once tumors reached a mean tumor length of 50 mm, the mice were randomized into two study arms, based on tumor volume and body weight. Ten mice were treated with vehicle (PBS) and ten with gemcitabine (100 mg/kg) every 7 days. Animals were sacrificed if tumors reached 150 mm or severe drug side effects were evident.

**Figure 5:**
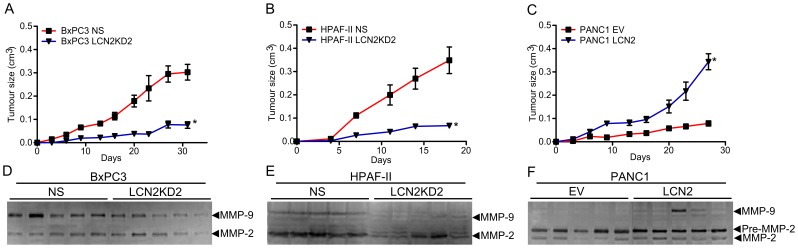
LCN2 promotes tumor growth and invasion *in vivo*. Growth curves of tumors formed by (A) BxPC3 NS and –LCN2KD2, (B) HPAF-II NS and –LCN2KD2, and (C) PANC1 EV and –LCN2 cells implanted subcutaneously in SCID mice. Gelatin zymography was perform on protein lysates isolated from (D) BxPC3 NS and –LCN2KD2, (E) HPAF-II NS and –LCN2KD2, and (F) PANC1 EV and –LCN2 xenografts (*denotes significance between KD and NS, or LCN2 and EV, p<0.05, student t-tests, n = 5).

### Immunohistochemistry

The development of the PanIN and PDAC tissue microarrays was reported previously [4]. Immunohistochemistry methodology was also previously described [20]. LCN2 antibody was used at 1∶2000 dilution following pepsin digestion. The relative LCN2 staining pattern and intensity were scored on a scale from 0 to 3 and multiplied by the percentage of positive cells which were scored as follows: 1∶1–25%; 2∶26–50%; 3∶51–75%; 4∶76–100%. Thus, the range of scores was between 0–12. The slides were scored independently by LL and BB, a board-certified pathologist. Blood vessel counting method as described before [20]. Briefly, blood vessels were identified by immunostaining with murine CD31 antibody. Blood vessels were scanned under 40x and 100x magnification, and were counted for discrete vessel formation. The number of discrete vessels found within a 100x field was counted five times for each xenograft assessed.

**Figure 6:**
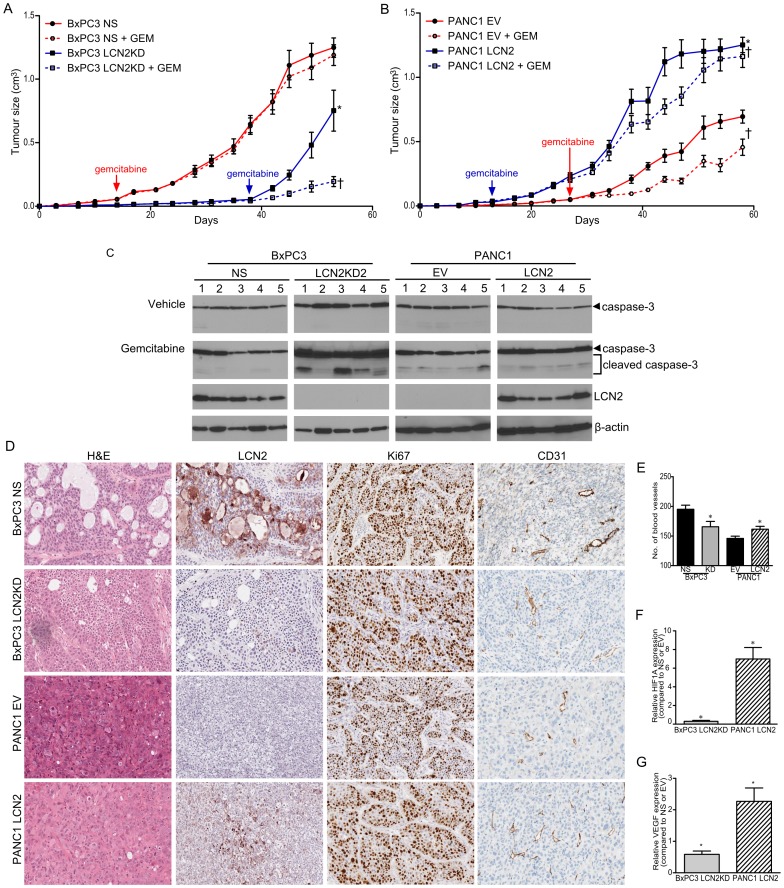
LCN2 promotes resistance to gemcitabine and angiogenesis. Effect of gemcitabine on the growth of tumors formed by (A) BxPC3-NS and –LCN2KD2 cell lines and (B) with PANC1-EV and –LCN2 cell lines [*denotes significance between vehicle treated cell lines (n = 10 per group, p<0.0001, mixed model multiple regression) †denotes significance between vehicle and gemcitabine treated mice injected with BxPC3 LCN2KD2 (n = 10 per group, p = 0.0003, mixed model multiple regression)]. (C) Protein lysates isolated from BxPC3 and PANC1 xenografts were assayed for caspase-3 cleavage after gemcitabine treatment (n = 10). (D) Representative histological images of xenografts formed by BxPC3 NS and –LCN2KD2, and PANC1-EV and –LCN2 cells after H&E, and immunostaining for LCN2, Ki67, and murine CD31. (E) Vascular density in five hot spots at high magnification in the BxPC3 NS and –LCN2KD2, and PANC1-EV and –LCN2 xenografts. The mRNA expression of (F) HIF1A and (G) VEGF in the BxPC3 and PANC1 xenografts. Gene expression was compared between KD and NS, or LCN2 and EV. [*denotes significance between KD and NS, or LCN2 and EV (n = 20; p<0.05, student t-test)].

### Microarray Analysis

The effect of LCN2 suppression was evaluated by transcriptional profiling in the BxPC3 cell lines and xenografts using the Illumina HumanHT-12 v4 array (Illumina, San Diego, CA). The data were normalized using log_2_-transformation and quantile normalization. Moderated paired t-tests were used to compare samples and controls. Common differences in fold changes that were ≥1.5-fold were included in our analyses carried out using SAS v9.2. GO term analysis of genes significantly associated with LCN2 using microarray analysis was performed using the Database for Annotation, Visualization and Integrated Discovery (*DAVID*) v6.7.

### Statistical Analysis

LCN2 immunostaining of tissue microarrays were analysed using the Kruskal-Wallis one-way ANOVA, data as indicated were analyzed using ANOVA, and student t-test using GraphPad Prism 5 (La Jolla, CA). Data are presented as the means ± SEM. P values <0.05 were considered significant. The tumor volume measured over time was analyzed after log-transformation using the mixed effect regression model using the LCN2 (KD vs. NS) and the treatment (Gem vs. PBS) as explanatory variables using R 2.9.1 software.

## Results

### LCN2 Expression in Multi-stage Pancreatic Duct Cell Carcinogenesis

To determine the association between LCN2 immunoreactivity and PDAC pathogenesis, LCN2 expression was assessed by immunohistochemistry using tissue microarray constructed to include normal (n = 31), pancreatic intraepithelial neoplasia (PanIN)-1 (n = 22), PanIN-2 (n = 13), PanIN-3 (n = 13), and PDAC (n = 82) [4]. In normal pancreas, LCN2 immunostaining was weak to moderate and confined to the duct cells within the cytoplasm and membrane, (mean score: 2.77±0.42; Fig. 1A). The staining pattern intensified in the small/medium sized ducts, and lumen of the larger ducts of high grade PanINs. The mean scores were 3.95±0.55 for PanIN-1, 8.00±0.88 for PanIN-2, and 8.27±1.01 for PanIN-3. All of the PDAC tissue samples stained positive for LCN2 expression (mean score: 5.93±0.33). Significant differences in staining were observed between normal pancreas and PanIN-2 and -3 lesions, as well as normal compared to PDAC (p<0.001).

### LCN2 Expression in PDAC Cell Lines

After determining LCN2 staining in PanIN and PDAC samples, we next wanted to assess LCN2 mRNA expression in 21 PDAC cell lines. 80% of the cell lines displayed elevated expression compared to the normal H6c7 cell line (Fig. 1B). However, MiaPaca2, PANC1, PK1, and PK8 PDAC cell lines showed minimal or no LCN2 expression compared to H6c7 cells (Fig. 1C). By Western blot, protein expression levels were concordant with mRNA levels in the majority of the cell lines.

### Knockdown and Overexpression of LCN2 in PDAC Cell Lines

We previously reported an increased LCN2 expression after KRAS^G12V^ expression in H6c7 cells [4]. This expression was maintained in the tumor cell line, H6c7KrT established from a tumor that developed subcutaneously after implantation of H6c7KRAS^G12V^ cells in SCID mice [4]. LCN2 mRNA expression was 10- and 2-fold higher in H6c7KRAS^G12V^ and H6c7KrT cells compared to the vector control H6c7-pBp cells [4]. Three separate shRNA sequences were used to downregulate LCN2 expression in H6c7KrT cells, resulting in reduction of LCN2 mRNA levels by 76%, 92%, and 65%, as compared to the non-silencing (NS) shRNA (Fig. 2A). Protein lysates and conditioned media also showed concordant reduction in LCN2 protein expression (Fig. 2B).

The LCN2KD2 shRNA sequence was selected for subsequent experiments as it yielded maximal knockdown of LCN2 expression. Stable transduction of LCN2KD2 and NS shRNA into the BxPC3 and HPAF-II cell lines decreased LCN2 mRNA expression by 78% and 87%, respectively (Fig. 2C). We stably transduced the low LCN2 expressing PANC1 cells with a LCN2 expression construct or a control empty vector (EV). Q-PCR and immunoblot analyses confirmed the overexpression (Fig. 2D). Secreted production of LCN2 was consistent with intracellular protein levels. Modifying LCN2 expression in PDAC cell lines did not alter changes in cell growth rate (Fig. S1A–C).

### LCN2 Improves Adhesion and Invasion of PDAC Cells

LCN2 has been reported to mediate attachment to the basement membrane [21]. To investigate if LCN2 promotes adhesion in PDAC, LCN2 was suppressed in the H6c7KrT, BxPC3, and HPAF-II cell lines. Knocking down LCN2 decreased attachment of cells on fibronectin and collagen coated plates compared to the NS control (p<0.05; Fig. 3A–B). LCN2 overexpression increased adhesion in PANC1 cells compared to EV control (p<0.05). Thus, LCN2 contributes to the adhesion of PDAC cells on fibronectin and collagen I substrata.

The binding of LCN2 to MMP-9 has been shown to prolong its enzymatic activity thereby enhancing invasion [22]. Invasion assays were performed to determine if LCN2 downregulation in H6c7KrT, BxPC3 and HPAF-II cells attenuated invasion through Matrigel and/or collagen IV coated membranes. LCN2 downregulation in H6c7KrT, BxPC3 and HPAF-II cells decreased invasion through Matrigel and/or collagen IV coated membranes. Each shRNA construct significantly diminished invasion by H6c7KrT cells through Matrigel by 71%, 77%, and 56%; and collagen IV by 72%, 80%, and 70%, respectively (p<0.01; Fig. 3C). Knocking-down LCN2 in the BxPC3 and HPAF-II cell lines significantly reduced invasion through collagen IV by 60% and 70%, respectively (p<0.05). However, suppression of LCN2 affected only the ability of the HPAF-II cell line to invade through Matrigel (p<0.01; Fig. 3D). Elevated LCN2 expression in PANC1 cells enhanced invasion through both substrata (p<0.05).

Gelatin zymography was performed to assess the interaction between LCN2 and MMP9. Conditioned media was collected from the H6c7KrT, BxPC3, HPAF-II, and PANC1 cell lines to assess MMP-9 activity after LCN2 modification. MMP-9 expression levels remained consistent after LCN2 modification (Fig. S1D). LCN2 downregulation in H6c7KrT, BxPC3 and HPAF-II cell lines decreased MMP-9 activity by 30%, 66% and 88%, respectively (Fig. 3E, F, S1E). LCN2 expression in the PANC1 cell line caused a 5.4-fold increase in MMP-9 activity (Fig. 3F). However, altering LCN2 expression does not affect migration of PDAC cell lines (Fig. S1F). Thus, LCN2 contributes to the invasiveness of PDAC cells by promoting MMP-9 activity.

### LCN2 Enhances Gemcitabine Resistance in PDAC Cells *in vitro*

Gemcitabine is used as a first-line chemotherapeutic agent in PDAC [23]. To determine if LCN2 promotes survival in PDAC, BxPC3, HPAF-II, and PANC1 cell lines were treated with gemcitabine or vehicle for 72 hours and assessed for cell viability by propidium iodide (PI) exclusion. Knocking-down LCN2 in the BxPC3 and HPAF-II cell lines significantly increased the number of PI-positive cells indicating cell death, while LCN2 overexpression in PANC1 cells conferred increased resistance to gemcitabine (p<0.05, Fig. 3G). Immunoblot analysis of cleaved caspase-3 validated the flow cytometry results. Depleting LCN2 expression increased caspase-3 cleavage by two-fold in gemcitabine treated BxPC3 and HPAF-II cell lines, while LCN2 expression in PANC1 reversed this effect (Fig. 3H). No significant differences in the expression of anti-apoptotic proteins Bcl-xL and Bcl-2, and pro-apoptotic proteins Bad, Bax, and Bim were observed after altering LCN2 expression. The half maximal inhibitory concentrations (IC50) of gemcitabine dependence on LCN2 were investigated. The IC50 concentrations of the control BxPC3, HPAF-II, and PANC1 were 10 µM, 50 µM, and 20 µM, respectively. Knocking down LCN2 in the BxPC3 and HPAF-II cell lines reduced the IC50 concentrations to 5 µM and 20 µM, respectively. Whereas, expression of LCN2 in PANC1 increased the IC50 to 50 µM of gemcitabine (n = 5; Fig. S1F–H).

### LCN2-associated Global Transcriptional Changes

Several studies have identified LCN2 as an upregulated gene in cancer. However no studies have yet examined the effect of LCN2 on gene expression. To examine how LCN2 affects gene expression in PDAC cell lines, transcriptional profiling was performed on the BxPC3 cell lines and xenografts. LCN2 expression upregulated the expression of 623 genes (Table S1) and downregulated the expression of 538 genes (Table S2). The putative LCN2 target genes were annotated to GO biological processes and were significantly enriched for processes involved in apoptosis (28/623; p = 0.008), cell cycle (32/623; p = 0.02), and adhesion (14/623, p = 0.02). The downregulated genes annotated to GO biological processes were significantly enriched for genes involved in apoptosis (36/538; p = 0.004).

The genes involved in apoptosis were analysed and revealed 57% of the upregulated genes were involved in survival, and 67% of the downregulated genes were pro-apoptotic (Fig. 4A). To validate this we performed Q-PCR in the BxPC3, HPAF-II, and PANC1 cell lines. The pro-apoptotic gene AIFM1 was identified to have higher expression in the BxPC3 and HPAF-II cell lines after LCN2 was knocked-down (Fig. 4C). Whereas, expressing LCN2 in PANC1 cells enhanced expression of anti-apoptotic genes BIRC2, FAIM, and MCL-1 compared to the control (p<0.05; Fig. 4D–F).

Additionally, the genes enriched for attachment were examined (Fig. 4B). 44% of the genes promoted cell to cell attachment, whereas the remaining genes positively regulated cell to ECM adhesion. Q-PCR validation demonstrated that expressing LCN2 in the PANC1 cell lines promoted expression of LAMAC2, MMP-7, CDH11, and ITGA2 (p<0.05; Fig. 4G–J). Whereas depleting LCN2 expression in the BxPC3 and HPAF-II cell lines downregulated expression of MMP-7 and CDH11 (p<0.05; Fig. 4H, I). We identified that LCN2 enhances expression of genes annotated to adhesion and survival in PDAC.

### LCN2 Promotes Tumorigenicity in the Xenograft Model

The expression of LCN2 has been demonstrated to enhance breast tumour formation and progression [13]. To determine how LCN2 affects tumorigenicity in PDAC, BxPC3, HPAF-II, and PANC1 cells were implanted subcutaneously into SCID mice. Knocking-down LCN2 in both BxPC3 and HPAF-II cells significantly reduced tumor growth when compared to the NS xenografts (p<0.01; Fig. 5A–B). Consistently, PANC1 LCN2 cells had markedly increased tumor growth compared to the EV xenografts (p<0.01; Fig. 5C). Gelatin zymography was employed to examine the effects of suppressing or overexpressing LCN2 on MMP-9 activity in PDAC xenografts. Diminishing LCN2 expression reduced MMP-9 activity by 35% and 79% in BxPC3 and HPAF-II xenografts, respectively (Fig. 5D–E, S2A). Whereas, in PANC1 xenografts there was little MMP-9 activity and LCN2 expression promoted MMP-9 activity in half of xenografts assessed (Fig. 5F). Together, LCN2 expression influences tumourigenicity *in vivo* and promotes MMP-9 activity in PDAC cell lines that highly express LCN2.

### LCN2 Promotes Gemcitabine Insensitivity in Resistant PDAC Cells *in vivo*

Since LCN2 has been demonstrated to promote survival in PDAC and several cancer cell line models *in vitro*, we wanted to determine if LCN2 would have an effect on gemcitabine sensitivity of PDAC *in vivo* [10], [17]. Tumor bearing mice were treated with vehicle (PBS) or 100 mg/kg gemcitabine once every seven days. BxPC3 is inherently insensitive to gemcitabine, whereas PANC1 is sensitive *in vivo* [24]. Gemcitabine treated BxPC3 NS mice showed no change in tumor growth compared to the vehicle treated mice. Attenuating LCN2 expression in BxPC3 cells reduced tumor growth (p<0.0001) and increased sensitivity to gemcitabine (p = 0.0003; Fig. 6A; Table S3). In PANC1 cells, LCN2 expression enhanced tumor growth (p = 0.00035; Fig. 6B), but was not correlated with increased resistance as gemcitabine (p<0.00001). Knocking down LCN2 in BxPC3 xenografts had increased cleaved caspase-3 activity by over 5-fold after treatment with gemcitabine compared mice bearing control xenografts (Fig. 6C, S2B; p<0.0001). Whereas, expressing LCN2 in the PANC1 xenografts reduced cleaved caspase-3 activity by 30% (p = 0.035). Assessment of LCN2 immunostaining revealed that the BxPC3 xenografts maintained the expression of the shRNA targeted against LCN2, and the PANC-1 xenografts retained expression of the LCN2 construct (Fig. 6D). Furthermore, Ki67 immunostaining did not demonstrate any differences in proliferation between high and low LCN2 expressing xenografts. Thus, LCN2 promotes gemcitabine resistance in insensitive lines.

#### LCN2 promotes angiogenesis

HIF1A was identified as one of the significantly upregulated genes in the microarray analysis which prompted us to assess the vascularity and VEGF expression in the BxPC3 and PANC1 xenografts [25]. The quantification of CD31 positive blood vessels revealed that knocking-down LCN2 in BxPC3 cells decreased expression of HIF1A and VEGF, and vascularity by 15% (Fig. 6E–G). Whereas expressing LCN2 in PANC1 cells increased vascularity by 11% and elevated expression of these angiogenic genes *in vivo* (p<0.05). We conclude that LCN2 promotes tumor growth, invasion, angiogenesis, and maintains resistance to gemcitabine in insensitive lines.

## Discussion

In the present study, the use of multiple modalities has provided a cohesive study into the function of LCN2 in PDAC and its pattern of expression during pancreatic carcinogenesis. We have shown that LCN2 expression is associated with the progression of PanIN lesions and PDAC. Through expression profiling studies, we have also demonstrated that LCN2 upregulates genes involved in survival, adhesion, and cell cycle, and downregulates pro-apoptotic genes. We have provided strong evidence that LCN2 promotes attachment, invasion, tumor growth, and gemcitabine resistance in multiple PDAC cell lines.

By modifying LCN2 expression we were able to demonstrate by gelatin zymography that it modulates MMP-9 enzymatic activity. Depleting LCN2 abrogates invasion through basement membrane substrata, Matrigel, and collagen IV by PDAC cells. Since MMP-9 is a collagenase, depleting LCN2 in the BxPC3 and HPAF-II cell lines attenuated invasion through collagen IV. However, invasion through Matrigel was hindered in the BxPC3 cell line only. Matrigel is composed of other extracellular matrix proteins besides collagen such as laminins and proteoglycans, and represents a more complex substratum. Therefore, altering LCN2 may elicit diverse invasive phenotypes in different PDAC cell lines. Our findings that LCN2 expression promotes MMP-9 activity are consistent with other cancer cell types. Depletion of LCN2 in colon [21], gastric [16], and breast cancer models [13], [26] diminishes MMP-9 activity thereby attenuating invasion. Furthermore, the presence of LCN2-MMP-9 complexes has been found in the urine of breast cancer patients and in tissue homogenates of gastric cancer patients [12], [15]. Clinically, detection of this complex can be used as a diagnostic predictor for breast cancer, has been associated with poor prognosis in gastric cancer, and was linked to depth of tumour invasion in esophageal cancer [12], [15]. The presence of LCN2 protects MMP-9 from autodegradation which prolongs its activity [22]. Hence, the upregulation of LCN2 promotes the invasive progression of PDAC.

LCN2 stimulates tumour growth *in vivo*. In this study, enhancing LCN2 expression in PANC1 cells markedly increased tumor growth, whereas suppressing its expression in BxPC3 cells reduced tumor growth rate. Similar findings have been found in several breast cancer studies that have demonstrated the critical role of LCN2 expression in the aggressive growth of tumours [12], [13], [26], [27] and the dissemination of metastases [13], [26], [27]. The promotion of tumour growth has been associated with enhanced VEGF expression in endometrial cancer [28]. Moreover, we identified that LCN2 promoted HIF1A and VEGF expression in several PDAC cell lines which contributed to an increase in vascularity. Together, our data supports the association of LCN2 expression and aggressive tumour growth by stimulating angiogenesis through the upregulation of HIF1A and VEGF.

LCN2 has been shown to suppress apoptosis in thyroid, lung, and breast cancers, thus we wanted to ascertain if LCN2 promoted survival in PDAC [10], [17]. Low LCN2 expressing cell lines had increased cleaved caspase-3 and PI-inclusion after gemcitabine treatment *in vitro*. Furthermore, depleting LCN2 in the gemcitabine resistant BxPC3 cell line restored sensitivity and increased caspase-3 cleavage *in vivo*. Several recent studies have implicated LCN2 as an anti-apoptotic protein by blocking the activation of caspase-9 through Bim [10], [17]. Though changes in Bim expression were not noted, we did find differences in mRNA expression of pro-apoptotic AIFM, and anti-apoptotic BIRC2, FAIM, and Mcl-1. In addition, expression of LCN2 promoted expression of genes involved in cell survival. In summary, we provide evidence utilising several cell lines that LCN2 promotes tumorigenicity in PDAC by enhancing invasion, tumour growth, angiogenesis, and gemcitabine resistance.

Contradicting our findings and other studies, Tong *et al*. suggested that LCN2 acts as a tumor suppressor gene in PDAC [25]. They reported that using a single shRNA to downregulate LCN2 expression in the high LCN2 expressing BxPC3 cells led to increased invasion and attachment. While forced LCN2 expression in MiaPaca2 decreased tumour size, metastatic spread, VEGF expression, and microvascular density [25]. In addition, Tong and colleagues observed that LCN2 did not confer any protection against gemcitabine after 48 hours of *in vitro* treatment, and they did not study gemcitabine sensitivity *in vivo* [25]. We also observed no differences within 48 hours of treatment *in vitro*, but after 72 hours of gemcitabine treatment we found LCN2 expressing cell lines were more chemoresistant which is consistent with other experimental models where LCN2 acts as an anti-apoptotic protein [10], [17]. Contrary to our findings, Tong et al. reported that LCN2 decreases VEGF expression, HUVEC tubule formation, and microvascular density in MiaPaCa2 xenografts [25]. In our study, we assessed VEGF and HIF1A expression in three PDAC cell lines since these genes were identified to be highly upregulated in the microarray. We observed changes in vascularity after altering LCN2 expression in the BxPC3 and PANC1 xenografts which validates the expression changes observed in HIF1A and VEGF. Furthermore, we investigated how LCN2 promotes tumourigenicity in three PDAC cell line xenograft models, whereas Tong and colleagues assessed this effect in the MiaPaCa2 cell line only [17]. Despite the many studies that have highlighted the interaction between LCN2 and MMP-9 which promotes invasion [12]–[14], [22], [29], Tong et al. did not explore this key interaction in their study [25]. The lack of concordance between our findings and the Tong *et al*. study may be partially attributed to differences in cell lines, experimental design, and differentiation state [25], [30], [31].

PDAC is one of the most fatal cancers with a very poor prognosis [1] and we have provided evidence on the importance of *LCN2* in contributing to aggressive and drug resistant phenotypes. Currently CA19-9 is the most commonly used serum biomarker to diagnose PDAC [23]. Recently, elevated serum LCN2 has been identified in acute pancreatitis patients [7]. Increase in LCN2 expression has been reported in renal injury, inflammation, and other cancer types [5]. The elevation of LCN2 in these numerous conditions indicates that regulation of its expression is varied which reflects its multi-faceted function. The expression of LCN2 was found to be ubiquitously expressed in PDAC patient samples and its elevated expression was associated with high-grade lesions. LCN2 has been described to be a potential biomarker for cancer progression as it has been found in the urine of breast cancer patients and in the serum of PDAC patients [12], [32]. Thus, LCN2 may represent a new biomarker for early diagnosis, prognostication and therapeutic targeting in PDAC.

## Supporting Information

Figure S1 **Cell growth curves for (A) BxPC3 NS and –LCN2KD2, (B) HPAF-II NS and –LCN2KD2, and (C) PANC1 EV and –LCN2 (n = 3).** MMP-9 gene expression and activity were assessed in BxPC3, HPAF-II, and PANC1 cells after modulating LCN2 expression by (D) Q-PCR and (E) gelatin zymography, respectively (*denotes significance p<0.05, student’s t-test, n = 3). (F) Migration was assessed at 0, 24, and 48 hours after the scratch was made on confluent BxPC3 NS and –LCN2KD2, HPAF-II NS and –LCN2KD2, and PANC1 EV and –LCN2 cells. The percentage of cells migrating in the wound are as noted (n = 3). IC50 concentrations were assessed in (G) BxPC3 NS and –LCN2KD2, (H) HPAF-II NS and –LCN2KD2, and (I) PANC1 EV and –LCN2 by MTS assay (n = 5).
(TIF)

Figure S2
(A) Gelatin zymography was performed on protein lysates extracted from BxPC3 NS and –LCN2KD2, HPAF-II NS and –LCN2KD2, and PANC1 EV and –LCN2 xenografts (* denotes significance p<0.05 between the test and control samples, student’s t-test, n = 5). (B) Cleaved caspase-3 was assessed in vehicle and gemcitabine treated BxPC3 and PANC1 xenografts. Cleaved caspase-3 activity was normalised against the β-actin loading control (* denotes significant differences between the vehicle and gemcitabine treatment, † denotes significance between vehicle treated NS and gemcitabine treated LCN2KD2 samples, 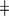 denotes significance between gemcitabine treated control and test samples, § denotes significance between vehicle treated LCN2 expressing and gemcitabine treated EV samples, one-way ANOVA and Bonferroni’s post hoc tests, n = 10).(TIF)

Table S1 **LCN2 upregulated genes according to their annotated functions.**
(DOC)

Table S2 **LCN2 downregulated genes according to their annotated functions.**
(DOC)

Table S3 **Differences in log growth rates after LCN2 modification and gemcitabine treatment.** Differences in log growth rates between each of the eight comparisons are noted.
(DOC)

Table S4 **Individual primer sets used in QPCR.**
(DOC)
