# Supplementary figures and images for: Lipocalin2 Promotes Invasion, Tumorigenicity and Gemcitabine Resistance in Pancreatic Ductal Adenocarcinoma
Source: PLoS One. 2012 Oct 4;7(10):e46677. doi: 10.1371/journal.pone.0046677 (PMC3464270; doi:10.1371/journal.pone.0046677)

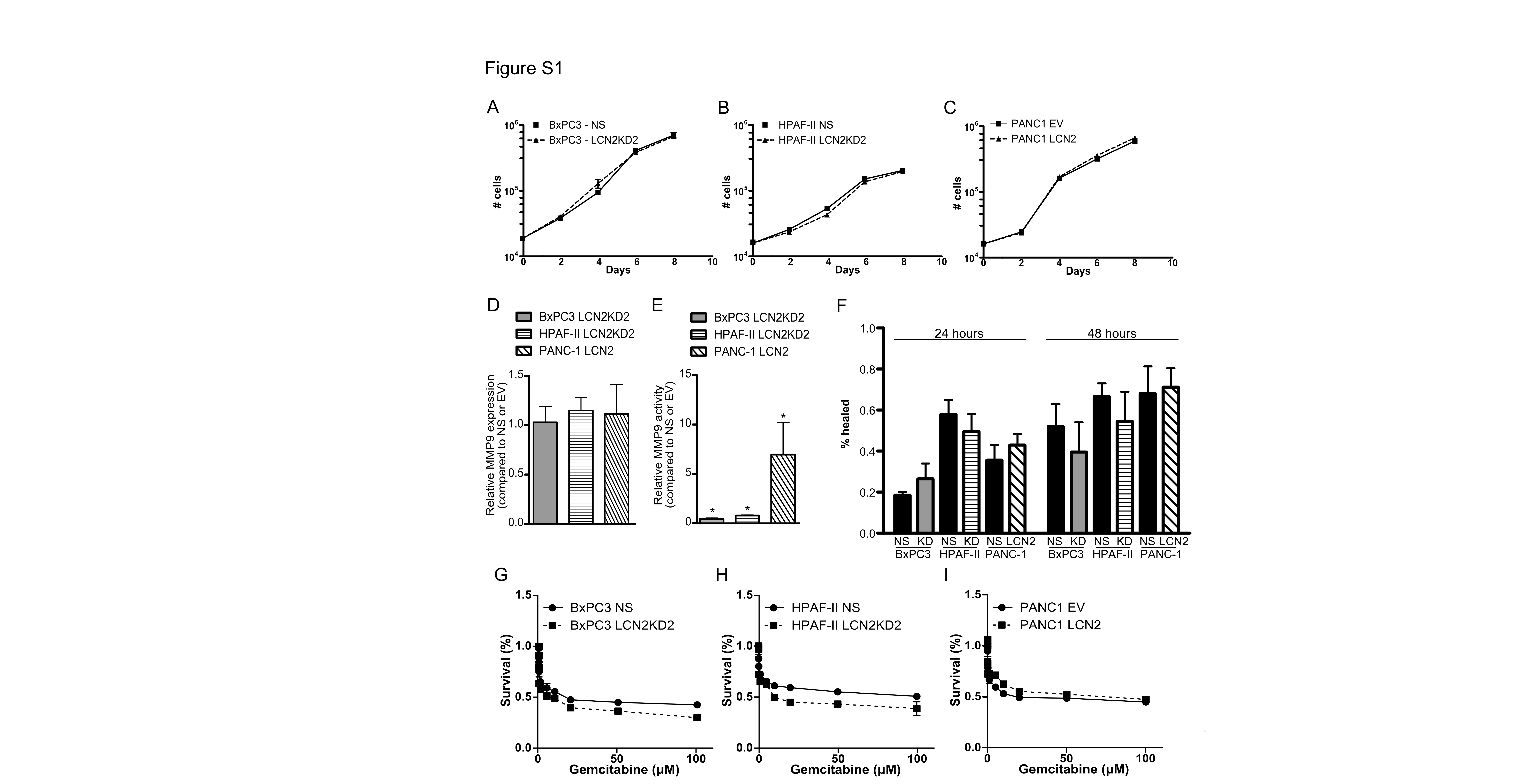

Supplement: Figure S1 — Cell growth curves for (A) BxPC3 NS and –LCN2KD2, (B) HPAF-II NS and –LCN2KD2, and (C) PANC1 EV and –LCN2 (n = 3). MMP-9 gene expression and activity were assessed in BxPC3, HPAF-II, and PANC1 cells after modulating LCN2 expression by (D) Q-PCR and (E) gelatin zymography, respectively (*denotes significance p<0.05, student’s t-test, n = 3). (F) Migration was assessed at 0, 24, and 48 hours after the scratch was made on confluent BxPC3 NS and –LCN2KD2, HPAF-II NS and –LCN2KD2, and PANC1 EV and –LCN2 cells. The percentage of cells migrating in the wound are as noted (n = 3). IC50 concentrations were assessed in (G) BxPC3 NS and –LCN2KD2, (H) HPAF-II NS and –LCN2KD2, and (I) PANC1 EV and –LCN2 by MTS assay (n = 5). (TIF) [file pone.0046677.s001.tif]

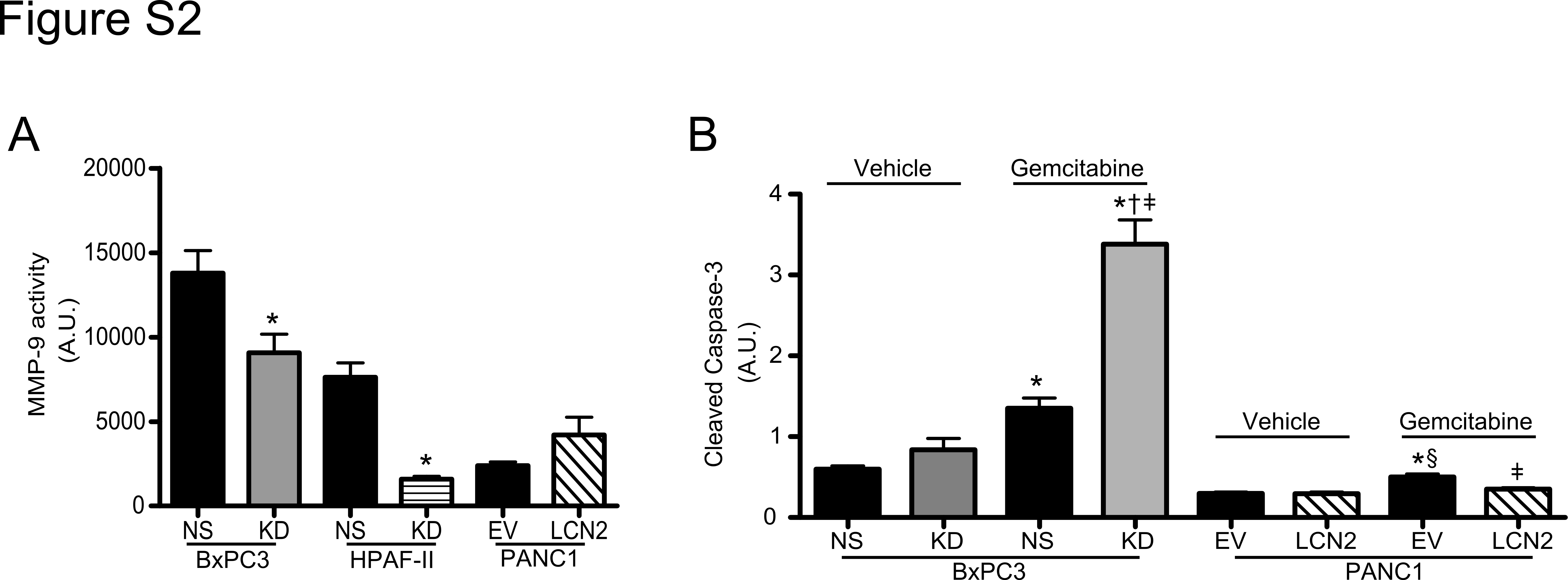

Supplement: Figure S2 — (A) Gelatin zymography was performed on protein lysates extracted from BxPC3 NS and –LCN2KD2, HPAF-II NS and –LCN2KD2, and PANC1 EV and –LCN2 xenografts (* denotes significance p<0.05 between the test and control samples, student’s t-test, n = 5). (B) Cleaved caspase-3 was assessed in vehicle and gemcitabine treated BxPC3 and PANC1 xenografts. Cleaved caspase-3 activity was normalised against the β-actin loading control (* denotes significant differences between the vehicle and gemcitabine treatment, † denotes significance between vehicle treated NS and gemcitabine treated LCN2KD2 samples, denotes significance between gemcitabine treated control and test samples, § denotes significance between vehicle treated LCN2 expressing and gemcitabine treated EV samples, one-way ANOVA and Bonferroni’s post hoc tests, n = 10). (TIF) [file pone.0046677.s002.tif]
