# Supplementary material for: Lipocalin2 Promotes Invasion, Tumorigenicity and Gemcitabine Resistance in Pancreatic Ductal Adenocarcinoma
Source: PLoS One. 2012 Oct 4;7(10):e46677. doi: 10.1371/journal.pone.0046677 (PMC3464270; doi:10.1371/journal.pone.0046677)
Supplement: Table S1 — LCN2 upregulated genes according to their annotated functions. (DOC) [file pone.0046677.s003.doc]

Table S1: LCN2 upregulated genes according to their annotated functions

| **Intracellular non-membrane bound (n=104; p<0.001):**  ABI2, Actr2, Aif1, Anln, APOBEC3F, ARID4B, bin1, birc2, BOP1, Casp3, CA5, CDK5RAP2, CEBPG, Ckap2, Cstb, CTNNAL1, CTNNB1, DDX21, DYNLT3, Eif3e, Fbxo5, FERMT1, Foxk1, FRMD6, fscn1, FXR1, GRWD1, gtf2f2, GTPBP4, Hells, HIF1A, HMGA1, HMGB1, HMGB1L1, Igf2bp2, ILK, ING2, INTS6, KIF11, KLK6, Krt14, Krt16, KRT17, KRT6A, KRT6B, KRTAP3-2, LCP1, LOR, LRRCC1, LYAR, MCM4, Mprip, MYC, MYCBP2, MYL1, NCAPG, ndrg1,NEB, NMD3, Nop56, NUP133, OASL, ORC6L, PARVB, pfn2, Phf2, PKP1, PLEK2, Polr1c, PRKRIR, Procr, psmc1, RABGAP1, RAD21, RAI14, Ran, rnasen, RPF2, rpl21, RPL21P14, Rpl23a, rpl28, RPL36A, RPS24, S100A9, SCEL, SGCE, SMARCA5, SMC4, SNCG, sntb2, Sprr1a, Sprr1b, Stat3, stom, suv39h2, TBCE, TMSB10, TNS3, TOP2A, Wdr12, XRN2, ZNF148 |
| --- |
| **Nucleus (n=64; p<0.001):**  actl6a, APOBEC3F, BOP1, Casp3, CCNA2, CCND1, CEBPG, cnot7, CPSF3, Cstb, CTNNB1, DDX21, DFFB, Eif3e, ercc2, Fbxo5, Foxk1, foxo1, FXR1, GRWD1, gtf2f2, GTPBP4, HIF1A, HMGA1, HMGB1, Hnrnph2, ING2, INTS1, INTS6, KLK6, KRT17, LOR, LYAR, mcl1, MCM4, MYC, NFIB, NMD3, Nop56, OASL, ORC6L, Phf2, Polr1c, PRKRIR, psmc1, Ran, rnasen, RPF2, RPL36A, RSRC1, S100A9, SMARCA5, Stat3, stk24, SYNCRIP, Taf2, TCEA1, TFDP1, TNS3, TOP2A, Wdr12, XRN2, YAP1, ZNF148 |
| **Protein folding (n=14; p=0.001):**  cct6a, Cct7, CCT8, CCT8P1, Dnaja1, FKBP11, FKBP1A, HSP90AA1, HSPD1, HSPE1, pdia6, ppiD, SEP15, TBCE |
| **Apoptosis (n=28; p=0.008):**  birc2, Card6, Casp3, CGB8, Ckap2, CSE1L, CTNNB1, ddit4, DFFB, ercc2, FAIM, FXR1, GJA1, Gjb6, HSPD1P6, HSPE1, LGALS7B, MADD, mcl1, MYC, NET1, PDCD10, PEG10, RAD21, rtn4, SLTM, TNFRSF6B, TOP2A |
| **Cell cycle (n=32; p=0.02):**  ADCY3, Aif1, Anln, CCNA2, CCND1, CDCA5, Ckap2, CKS2, CTNNB1, dtymk, Fbxo5, Hells, ILK, KIF11, LFNG, LRRCC1, MYC, NCAPG, PPP3CB, psmc1, RABGAP1, RAD21, Ran, SESN3, Skp2, SMC4, suv39h2, Taf2, TFDP1, UHRF1, ZAK, zc3hc1 |
| **Adhesion (n=14; p=0.02):**  CDH6, PCDHGA12, ITGA2, LY6D, PARVB, COL7A1, CDH11, col12a1, CD164, adam12, CTNNB1, DSG3, LAMC2, cdh26, Fat1, FERMT1, SPP1, lpp, PKP1, MMP7 |
