# Supplementary material for: Lipocalin2 Promotes Invasion, Tumorigenicity and Gemcitabine Resistance in Pancreatic Ductal Adenocarcinoma
Source: PLoS One. 2012 Oct 4;7(10):e46677. doi: 10.1371/journal.pone.0046677 (PMC3464270; doi:10.1371/journal.pone.0046677)
Supplement: Table S2 — LCN2 downregulated genes according to their annotated functions. (DOC) [file pone.0046677.s004.doc]

Table S2: LCN2 downregulated genes according to their annotated functions

| **Endoplasmic reticulum (n=43; p<0.001):**  ALG13, Agr2, AIFM1, BCAP31, CYP2F1, cyp2j2, cyp4b1, CYP4F12, DHCR24, DNAJC10, Dnase1l1, EBP, ephX1, ERGIC1, ern2, FAF2, fmo3, HAX1, HSD11B2, Lman2, lpcat4, LRMP, Man1b1, mettl7a, MMGT1, Pex16, PGAP3, PGRMC1, PPP1R15A, prkcsh, sec22c, sez6l2, SGK1, SGPP2, SIL1, Slc27a5, Ssr4, STIM1, stt3a, TRAPPC6A, UGT2B17, UGT2B7, VAMP7 |
| --- |
| **Membrane (n=44; p<0.001):**  abcc4, Adcy6, AIFM1, ATP6V0E1, atp6v1b1, Camk2n1, Celsr3, CHPT1, Clec2d, CLIC1, CUL5, CYP2F1, cyp2j2, cyp4b1, CYP4F12, dnajc4, EBP, ephX1, fmo3, FOS, Gbas, gcnt3, GDI1, GRIN1, HSD11B2, lamp2, Man1b1, PGRMC1, PICK1, POMGNT1, PON3, PPAP2A, ptprf, SCNN1B, SLC12A2, SLC1A3, SLC1A5, Slc26a6, stt3a, SYTL2, tspan15, Tspan31, UGT2B17, VSIG2 |
| **Mitochondria (n=56; p<0.001):**  ACADM, ACO2, ACOT13, ACSF2, AFG3L2, AIFM1, ALDH18A1, ATP5G2, atp5l, BCKDHA, Brp44l, Chchd2, CKB, Cox7b, CTSA, D2hgdh, dci, Dpysl2, etfB, FIS1, FOXRED1, gatm, Gbas, GRAMD4, HAX1, hsd17b4, IDH2, isoc2, mccc2, MRPL20, mRpL41, msrB2, MTCP1, NDUFA1, Ndufa2, Ndufa8, NIPSNAP1, PCCB, pck2, PICK1, PNKD, PRODH, pts, sdhD, SIRT5, SLC1A3, slc25a10, SLC25A23, SUOX, taz, TNFRSF19, TSC22D3, UCP2, Uqcr10, UQCRB, Uqcrh |
| **Apoptosis (n=36; p=0.004):**  AFG3L2, AIFM1, Ang, ATP6AP1, BCAP31, Casp4, DAPL1, DHCR24, DNASE2, FIS1, ern2, GAS1, HPRT1, klf11, KRT8P9, LTB, LYZ, Moap1, mRpL41, Mst4, Muc5ac, NGFRAP1, nme3, OPTN, PPP1R15A, pycard, SGK1, SLC5A8, SLK, taf9b, TBRG4, TICAM1, TNFRSF14, TNFRSF19, TNFSF10, TSC22D3 |
