# Supplementary material for: Lipocalin2 Promotes Invasion, Tumorigenicity and Gemcitabine Resistance in Pancreatic Ductal Adenocarcinoma
Source: PLoS One. 2012 Oct 4;7(10):e46677. doi: 10.1371/journal.pone.0046677 (PMC3464270; doi:10.1371/journal.pone.0046677)
Supplement: Table S3 — Differences in log growth rates after LCN2 modification and gemcitabine treatment. Differences in log growth rates between each of the eight comparisons are noted. (DOC) [file pone.0046677.s005.doc]

Table S3: Differences in log growth rates after LCN2 modification and gemcitabine treatment

| Comparison | Difference in growth rate (log values) | p-value |
| --- | --- | --- |
| BxPC3 NS: Gem. vs Vehicle | -0.02736 | 0.7816 |
| BxPC3 KD: Gem vs Vehicle | 0.5168 | 0.0003 |
| Vehicle: BxPC3 KD vs NS | -0.5814 | <0.0001 |
| Gem.: BxPC3 KD vs NS | -0.03648 | 0.8147 |
| PANC1 EV: Gem. vs Vehicle | -0.026 | 0.00002 |
| PANC1 LCN2: Gem. Vs Vehicle | -0.028 | <0.00001 |
| Vehicle: PANC1 LCN2 vs EV | 0.02 | 0.00035 |
| Gem.: PANC1 LCN2 vs EV | 0.019 | 0.0038 |
