# Supplementary material for: Lipocalin2 Promotes Invasion, Tumorigenicity and Gemcitabine Resistance in Pancreatic Ductal Adenocarcinoma
Source: PLoS One. 2012 Oct 4;7(10):e46677. doi: 10.1371/journal.pone.0046677 (PMC3464270; doi:10.1371/journal.pone.0046677)
Supplement: Table S4 — Individual primer sets used in QPCR. (DOC) [file pone.0046677.s006.doc]

Table S4: Individual primer sets used in QPCR

| Gene name | Forward | Reverse |
| --- | --- | --- |
| *RPS13* | gttgctgttctaaagcatcttg | aatatcgagccaaacggtgaa |
| *TBP* | gggcattatttgtgcactgaga | tagcagcacggtatgagcaact |
| *ACTB* | tcctaaaagcaccccacttct | gggagaggactgggccatt |
| *LCN2* | ggtgttcttcaagaaagtttctcaaa | tccgaagtcagctccttggt |
| AIFM | gaaagacggcaggaaggtaga | gccaactcaacattgggct |
| BIRC2 | ttttcccaggtccctcgtatc | ccaatctgacaagatcgtgct |
| FAIM | ttgctttaagtgacggagtcc | gtctttgcagctccaacatagaa |
| MCL-1 | tgcttcggaaactggacatca | tagccacaaaggcaccaaaag |
| VEGF | tctacctccaccatgccaagt | gcgctgatagacatccatgaac |
| HIF1A | atccatgtgaccatgaggaaatg | tcggctagttagggtaacttc |
